# Crystal property and spectroscopic investigation on electro-optic and physico-chemical properties of NLO crystal; 3-(3,4-Dihydroxyphenyl)-L-Alanine

**DOI:** 10.1016/j.heliyon.2019.e03055

**Published:** 2019-12-24

**Authors:** D. Vidhya, S. Ramalingam, S. Periandy, R. Aarthi

**Affiliations:** a Department of Physics, A.V.C. College, Mayiladuthurai, Tamilnadu, India; b KMCPGS, Puducherry, India; c Department of Physics, ST. Theresa's College of Arts and Science, Tharangambadi, Tamilnadu, India

**Keywords:** Nonlinear physics, Optics, Organic chemistry, 3-(3,4-Dihydroxyphenyl)-L-Alanine, Birefringence, Laser damage threshold, CT complex, Electrophilic, Non-superposable, NLO activity

## Abstract

In this attempt, in order to obtain high-quality NLO crystal, organic compound; 3-(3,4-Dihydroxyphenyl)-L-Alanine crystal was fabricated. The organic-composite crystal was characterized by crystallographic and spectroscopic tools. The NLO supported parameters like crystal lattice (orthorhombic) and space group (*P*2_1_2_1_2_1_) examined and validated by XRD examination. The SHG test was carried out and SHG efficiency was calculated that1.29 and 1.35 times greater than solid KDP crystal. The laser damage threshold energy density was determined to be 14.51 GW/cm^2^. By the application of mulliken charge assignment, multiple dielectric cavities were found in crystal material which is able to process the high degree of birefringence gradient. The oscillating chemical potential movement was observed by examining chemical shift, among the core carbons of hexagonal ring and bridge carbons of chain. The chemical softness insists the binding viability of further ligand groups. The π and δ-conjugated interactive complex orbitals recognized on molecular site and participation in optical active mechanism was identified. UV-Visible transmission characteristics of crystal were studied and UV-Visible absorption on degenerate energy states was noted and its band gap energy was estimated. The CT complex of the present case was acknowledged to be COOH group and it causing crystal properties of current organic composite. The hyperactive polarizability was determined as 1775.05 × 10^−33^ esu and it was found to be five times greater than thiourea. The depletion energy between highly electrophilic zones and protonic zones was estimated to be ±5.241 e 2 causing permanent dielectric characteristics for the title organic composite. The non-superposable on the molecular mirror image was displayed and thereby optical ability was validated.

## 1 Introduction

The nonlinear optical characteristics crystal made by the different combination of organic and semi organic materials and their characterization towards its optical quality and optically induced mechanism is technologically fast growing and emerging field of current research. It plays vital role in fabrication of optical modulator, high frequency optical switch, frequency shifter and optical data storage devices. The customization of tuned hyper active non linear optical mechanism in high degree of dielectric organic crystals is the fundamental need of enhancing the second and third harmonic generation in the crystals is extremely important for both laser spectroscopy and laser processing technology. The improvement of developing optical efficiency even in IR and UV regions by doping of chiral and symmetrical molecules with optically active amino acids is very significant for producing parametric oscillation process [1].

The dielectric crystal made by organic materials have great consideration over two past decades as a result of superior optical, opto-electronic and photoactive characteristics due to their NLO hyperactivity and pure optical transparency [2, 3]. Amino acid doped organic material are always having wide and tuned sharp UV-optical band width by the symmetrical arrangement of conjugated π and δ-electron systems exhibit various photo-optic responses [4]. Non-toxic organic-dielectric crystal complex is usually customized by the well symmetric hexagonal structured organic [5] or semi organic compound adopted with Alanine which is enabled with high helical controlled non linear mechanism, chemical potential flexibility, high degree of non centro-symmetry, first order donor and acceptor stability and good optical transmittance render organic enhanced photoactive process [6].

1, 2-dihydroxybenzene known as catechol, is the ortho isomer in which two hydroxyl groups are consecutively substituted in the benzene ring is basically used as a precursor to organic crystals and pharmaceuticals [7]. The present compound was fabricated using the base; catechol and the crystal perfection were improved by the addition of Alanine (professional organic NLO composite beneath of amino acid category). The L-alanine is an important non-polar characteristic hydrophobic nature amino acid belongs to non-centrosymmetric space groups. Due to an asymmetric carbon core sequence, it is enabled as optically active [8]. Usually, optically resonance active amino acids acquire wide optical transparency range in UV-Vis spectral region. The combination of hexagonal frame substitution in alanine is a supreme electronic operated composite material for nonlinear optical devices [9].

Even though, the suitable combination of catechol and Alanine crystals is making huge impact on the fabrication of efficient organic NLO crystals, so far, no work is available to carry out the crystal growth and the characterization on such organic composite. Here, the present crystal was grown by simple slow evaporation technique and it was characterized morphologically, structurally, optically and spectroscopically using quantum computational methods.

## 2 Experimental methods

A mixture of 27.2 g (0.1 mole) of LI, 67.8 ml(0.6 mole) of 47% HBr, and the temperature of 28 ml (0.32 mole) of phenyl hydroxide was increased with magnetic stirrer and refluxed about 3 h and the deposited brown solution was allowed to be evaporated to a reddish syrup. It was dissolved in 30 ml of n-BuOAc and extracted twofold with 30 ml and 10 ml of H_2_O. The solution is kept in a refrigerator for 12 h and the corresponding powder was filtered.

For fabricating the present crystal compound, the L-Alanine and dihydroxy benzene were taken at 1:1 ratio and both are blended with one another by magnetic stirrer and boiled at 120 °C and also the combination was allowed to slow evaporation. The grown crystal was cleaved in its optical axis and it was allowed to characterize. The XRD pattern was recorded for raw sample of present case and the clear XRD pattern was determined for the analysis.

• The FT-IR spectral pattern was sequentially recorded using a Bruker IFS 66V with high resolution vib-rot spectrometer after making several scanning process in order to optimize the results.
• The FT-Raman spectral sequence was documented using Bruker spectrometer adopted with FT-IR instrument with Raman module equipped with a Nd:YAG laser source being opterated at 1.068 μm line width with 500 mW power.
• The high resolution ^1^HNMR and ^13^CNMR spectra were recorded using 300 MHz and 75 MHz NMR spectrometer with high magnetic gradient.
• The UV-Visible spectral prototype was recorded in solid phase in the region of 50 nm–700 nm, with the scanning interval of 0.50 nm, using the UV-1800 series instrument.

## 3 Computational methods

The present compound is the combination of amino acid and dihydroxy benzene and appeared to be helical structure which is to be optimized by hybrid methods of calculations. Here, all the structural parametric values are determined by B3LYP/6-311++G(d,p) method of computations and scanning process was performed to find out the optimized form of structure and it was validated to insert in crystal structure. The vibrational assignments were made on the characteristics wavenumber region which were calculated by B3LYP/B3PW91/6-311++G(d,p) methods and it was scaled in order to coincide the wavenumbers with observed spectral values. The molecular structure with minimized potential energy was depicted in which the molecular combination of alanine and dihydroxybenzene was clearly showed. The benzene structure was appeared at one plane and alanine was found to be helical form and other than benzene, the rest part of the molecule was become semicircle formation.

The structural parameters and mulliken charge assignment was calculated on the optimized structure using B3LYP/6-311++G(d,p) which are tabulated in ascending order. The chemical shift from ^1^H and ^13^C was computed in terms of TMs values and the restoring of chemical potential was determined. The FMO orbital interactions were monitored from which the non bonding orbital transitions were illustrated to find the chemical potential in non radiative transitions. The non linear process was studied by observing the hyper active polarizability taking place among molecular site. For analyzing molecular dipole moment, the electrostatic potential gradient was drawn from the grid points of distributed field using B3LYP/6-311++G(2d,2p) method of calculation.

## 4 Results and discussion

### 4.1 XRD analysis

**Figure 1:**
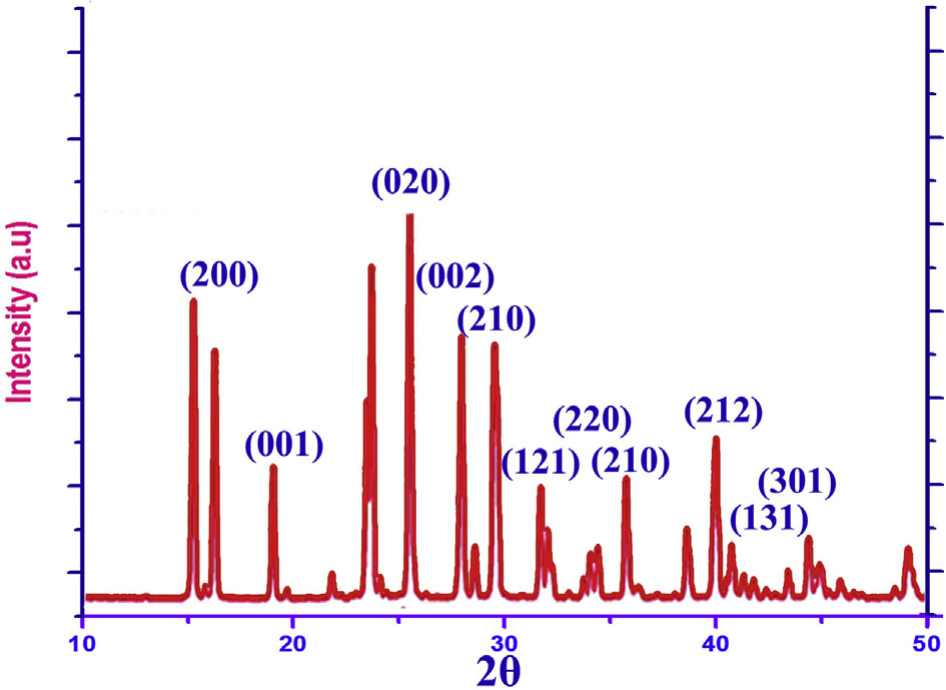
XRD spectral peaks of 3-(3,4-Dihydroxyphenyl)-L-Alanine.

The XRD spectral pattern was recorded for the present solid crystal sample and it is displayed in Figure 1. The clear XRD peak sequence was obtained with maximum intensity. The peaks were observed at 16°, 18°, 26°, 28°, 29°, 32, 36, 40°, 42° and 45° for (200), (001), (020), (002), (210), (121), (220), (210), (212) and (301) planes. Except some, all the XRD crust was observed with greatest diffraction intensity. This pattern of well distinct peaks is assigned to be orthorhombic crystal formation for present compound. Usually, below 20° of 2θ, the diffraction peaks not possible for organic crystals. But in this case, number of peaks was found to represent the orthorhombic crystal formation using present molecular structure; 3-(3,4-Dihydroxyphenyl)-L-Alanine. Although, the present organic compound would be in helical form, the perfect array of optimized structure formed well aligned orthorhombic crystal nature. Normally, the organic or semi-organic complex molecules are having flexibility of molecular design to construct well amalgamated crystal structure, here, the helical structure as well as hexagonal base of present case was found to be arranged in the form of three different sequence such as unequal axes at right angles and thereby orthorhombic crystal configuration was strongly shaped. In this case, the characteristic condition α = β = γ = 90° was satisfied from the obtained distinct peaks in different planes of the crystal system and C_S_ point group of symmetry was found to be adopted with the molecular system and thus, the *P*2_1_2_1_2_1_ space group was assigned according to the symmetry. From the above observation, it was clear that, the present crystal structure was customized on orthorhombic lattice and also confirmed that, the asymmetric core carbon sequence and non-centrosymmetric space groups induced NLO mechanism in the present crystal.

### 4.2 Crystal profile

The crystal parameters were observed from different parametric experimentation and are presented in Table 1. The base molecular structure for the crystal process and the grown crystal are displayed in Table 1. Here, two solvents were used to fabricate crystal by slow evaporation method and the melting point of crystal was determined to be 108 °C by raising its temperature in heating furnace. As per the bravais lattice rules, the space group of present crystal was measured to be *P*2_1_2_1_2_1_ whereas the point group of molecular structure was to be C_S_ symmetry which is recognized as noncentro-symmetric. The unit cell dimension was determined to be a = 6.321 Å, b = 13.629 Å and c = 5.864 Å which confirmed the orthorhombic lattice for the present organic crystal structure. This variable plane length of the crystal was ensured elevation of crystal unit cell repetition in different lattice plane sequence that strongly represents optical birefringence effect in the crystal. Correspondingly, the refractive indices of the crystal was determined to be n_1_ = 1.661, n_2_ = 1.874 and n_3_ = 1.519 respectively in three unequal optical axes which established the translation operation was invariably taking place in different planes inside the crystal system. The observed parametric indices including optical birefringence were well agreed with the literature [10, 11, 12]. For present crystallographic material, the birefringence gradient Δn was measured to be 0.189, was moderate and it is enough to produce double refraction modulation of a ray of light resultant in polarization. This present case was already found to be molecularly ordered organic complex which are having different refractive indices regarding to different crystallographic directions and was able to facilitate the Intrinsic Birefringence.

**Table 1:** Crystal parameters of 3-(3,4-Dihydroxyphenyl)-L-Alanine.

S. No.	Parameters	Values	Molecular/crystal structure
1	Space group	P2_1_2_1_2_1_	
2	Melting point	108 °C	
3	Unit cell		
	a	6.321 (Å)	
	b	13.629 (Å)	
	c	5.864 (Å)	
4	Refractive index		
	n_1_	1.661	
	n_2_	1.874	
	n_3_	1.519	
5	Birefringence (Δn) Kλ/t	0.189	
6	NLO efficiency	I^2ω^ = 15 X I^2ω^	
7	Transmittance	0.298–2.012 μm	
8	Complexity	210	
9	Volume	480.11 (Å)^3^	
10	Crystal lattice	Orthorhombic	
11	Crystal type	Enantiomorphic	
12	Dielectric constant (ε_r_) [1MHz]	50 °C = 4.821 & 60 °C = 5.112	

The Nonlinear optical property of present organic crystal was performed by Kurtz powder second harmonic generation (SHG) test by using popular Q-switched laser beam (Source-Nd: YAG laser). The crystal sample with same dimension was irradiated by laser (TEM_00_ mode locked) with input pulse of 6.8 mJ at λ = 1064 nm. The second harmonic signals were found at 348 mV and 389 mV from the present crystal sample with the optimum reference material of KDP at 265 mV from which the SHG efficiency was calculated to be 1.29 and 1.35 times greater than solid KDP crystal and the result is agreed well with the reported literature [13, 14]. The laser damage threshold energy density was determined by the mode of multiple shots damage and was identified to be 14.51 GW/cm^2^. When compared with standard KDP crystal, the experimental damage threshold energy value was found to be greater than KDP and other known organic crystals. The dielectric constant is the important factor which influences the NLO property of the organic material and composites directly. In this case, it was measured at 1 MHz frequency and it was to be 4.821 at 50 °C and 5.112 at 60 °C. The DC was increased much with respect to the temperature and decreased with frequency. The present crystal was Enantiomorphic and it was able to facilitate the interplanes with different refractive ability.

### 4.3 Structural property analysis

As per organic compositions of present case, the molecular geometry was arranged and thereby the crystal symmetry was spontaneously approved. All the structural parameters are depicted in the Table 2. In this case, according to the molecular arrangement, Hydrogen bond donor count, Hydrogen bond acceptor count and Rotatable bond count were calculated to be 4, 5 and 3 respectively. All the above parameters ensured the molecular planes and helical nature of the molecular setup and anisotropic characteristics and here, all the parameters were support for enforcement of crystalline quality. The Topological Polar Surface Area is the significant factor to make impact on crystal density and morphological integrity and it was 104 A^2^, for the crystal quality it should be greater than 100 A^2^ and here the observed value showed superiority of the crystal.

**Table 2:** Physical parameters of 3-(3,4-Dihydroxyphenyl)-L-Alanine.

Parameters	Values
Hydrogen bond donor count	4
Hydrogen bond acceptor count	5
Rotatable bond count	3
Topological Polar Surface Area	104 A^2^
Mono isotopic Mass	197.069
Exact Mass	197.069
Heavy Atom Count	14
Covalently-Bonded Unit Count	1
Ionic character	>1
‘N’ atom	14
MW	197.19
‘N’OHNH	5
‘N'violations	0
‘N'rotb	3
volume	172.00
Heteronuclear bonds counts	16
Homonuclear bond counts	8

The heavy atom count, Heteronuclear bonds counts and Homonuclear bond counts are consistently used to determine heterogeneity and anisotropic characteristics of the organic crystal material to provide Intrinsic Birefringence effect and they were found to be 14, 16 and 8 respectively. According to the observed values, the present organic composite is able to have efficient heterogeneity in molecular site of crystal compound and thereby the dielectric constant was improved for the betterment of crystalline excellence. The Covalently-Bonded Unit Count was 1 and ionic character was recognized to be >1 and these parameters provide large hyperpolarizability character for the molecule of crystal which enhanced the SHG efficiency of the composite.

### 4.4 Molecular geometry analysis

**Figure 2:**
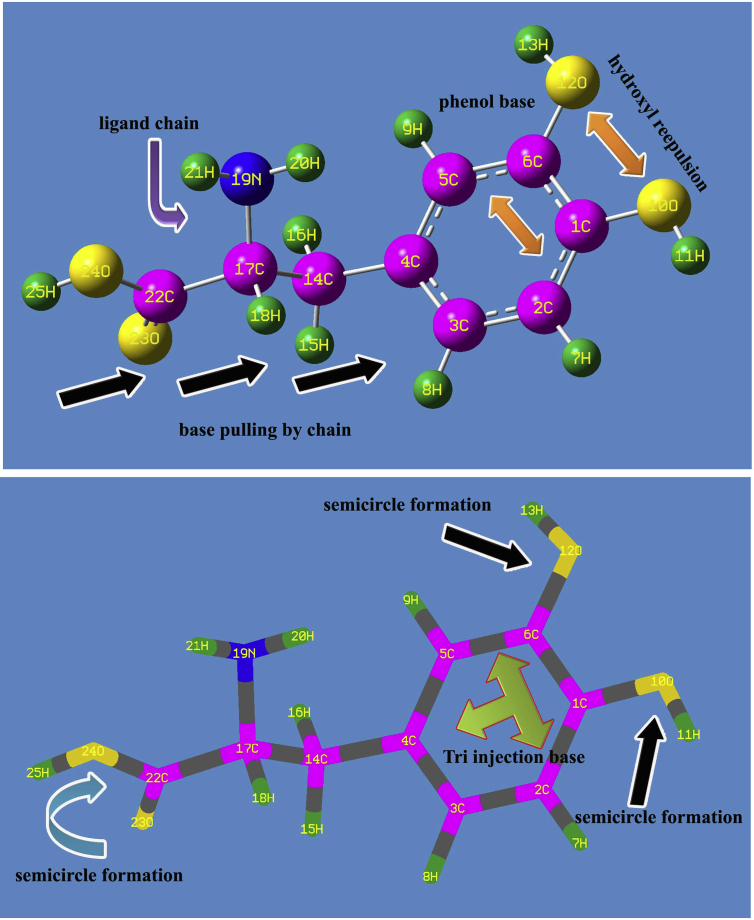
Molecular structure of 3-(3,4-Dihydroxyphenyl)-L-Alanine.

The title organic composite is composed by alanine and dihydroxy benzene by this application, the bond length and bond angle parameters are altered. The bond parameters alternation was made with respect to the electrochemical forces existed among the atomic site. Here, according to the standard value (1.391Å) for core CC of benzene ring [15], the tri-substituted benzene was found to be fractured multiply and it was evidenced by the change of bond lengths of C1–C2, C2–C3, C3–C4, C4–C5, C5–C6 and C1–C6 as 1.391, 1.394, 1.395, 1.400, 1.392 and 1.404 Å respectively as in the Table 3. The core CC of hexagonal ring system was differed from standard value from 0.001 to 0.013Å and it was well pronounced at C1, C6 and C4 by the substitutions. Other than core, the bond length of C4–C14, C14–C17 and C17–C22 were 1.512, 1.539 and 1.524Å respectively and these bond lengths were very much stretched up to 0.121Å due to the further addition of electronegative atoms in the chain. The bond length of O–H in phenol mode was less stretched up to 0.007Å than O–H in acid group as shown in Figure 2. Here, the alternative polarized and non polarized bonds in the chain produced heterogeneity molecular atmosphere to induce anisotropic ambiance in the crystal morphology.

**Table 3:** Optimized geometrical parameters for 3-(3,4-Dihydroxyphenyl Alanine.

Geometrical Parameters	Methods			
	HF	HF	B3LYP	B3PW91
	6-31+G (d, p)	6-311++G (d, p)	6-311++G (d, p)	6-311++G (d, p)
**Bond length(Å)**				
C1–C2	1.377	1.378	1.391	1.389
C1–C6	1.398	1.394	1.404	1.402
C1–O10	1.351	1.349	1.366	1.360
C2–C3	1.394	1.389	1.394	1.392
C2–H7	1.077	1.077	1.086	1.087
C3–C4	1.381	1.382	1.395	1.393
C3–H8	1.075	1.076	1.084	1.085
C4–C5	1.398	1.393	1.400	1.398
C4–C14	1.514	1.514	1.512	1.507
C5–C6	1.379	1.380	1.392	1.390
C5–H9	1.078	1.077	1.087	1.088
C6–O12	1.349	1.348	1.365	1.359
O10–H11	0.942	0.940	0.962	0.961
O12–H13	0.942	0.940	0.962	0.961
C14–H15	1.083	1.082	1.091	1.092
C14–H16	1.083	1.083	1.092	1.093
C14–C17	1.532	1.532	1.539	1.533
C17–H18	1.093	1.093	1.105	1.107
C17–N19	1.452	1.454	1.465	1.457
C17–C22	1.518	1.518	1.524	1.519
N19–H20	0.999	0.999	1.015	1.014
N19–H21	0.999	0.999	1.014	1.013
C22–O23	1.188	1.181	1.203	1.203
C22–O24	1.330	1.330	1.357	1.350
O24–H25	0.948	0.946	0.969	0.967
**Bond angle (°)**				
C2–C1–C6	118.9672	118.9017	118.9458	118.8839
C2–C1–O10	123.4797	123.3192	123.5306	123.5452
C6–C1–O10	117.5526	117.7786	117.5225	117.5698
C1–C2–C3	121.0747	121.1285	121.0673	121.1617
C1–C2–H7	119.3046	119.1958	119.0456	118.9879
C3–C2–H7	119.6201	119.674	119.8849	119.8481
C2–C3–C4	120.564	120.5821	120.6259	120.5899
C2–C3–H8	119.2667	119.1059	119.2983	119.3458
C4–C3–H8	120.1689	120.3092	120.072	120.0603
C3–C4–C5	117.9671	117.9194	117.9872	117.9663
C3–C4–C14	121.6896	121.2617	121.2026	121.1639
C5–C4–C14	120.3429	120.8181	120.8027	120.857
C4–C5–C6	121.8478	121.8774	121.8437	121.9302
C4–C5–H9	119.5739	119.449	119.4081	119.3535
C6–C5–H9	118.5744	118.6727	118.7466	118.7148
C1–C6–C5	119.5782	119.5897	119.5294	119.4673
C1–C6–O12	117.3926	117.6081	117.3813	117.4301
C5–C6–O12	123.0286	122.8021	123.0891	123.1025
C1–O10–H11	110.9844	110.6589	109.2927	109.0118
C6–O12–H13	111.0888	110.7019	109.2962	109.0088
C4–C14–H15	109.8769	109.5863	110.1127	110.2562
C4–C14–H16	109.8854	110.1047	110.3112	110.3277
C4–C14–C17	112.9872	113.1315	112.7857	112.5362
H15–C14–H16	107.025	106.9443	107.0739	107.1154
H15–C14–C17	108.5039	108.4128	108.2163	108.2961
H16–C14–C17	108.3708	108.4574	108.1457	108.126
C14–C17–H18	108.7759	108.6721	107.9734	107.7659
C14–C17–N19	111.1064	111.3668	110.9965	110.9411
C14–C17–C 22	110.6394	110.5569	110.7417	110.6466
H18–C17–N19	112.3937	112.2579	112.5633	112.736
H18–C17–C22	104.9252	104.9516	104.4348	104.0313
N19–C17–C22	108.8378	108.8556	109.9408	110.495
C17–N19–H20	110.5972	110.2803	109.7051	109.4412
C17–N19–H21	111.6626	111.2286	110.8773	110.8424
H20–N19 –H21	108.4834	108.0321	108.0393	107.9679
C17–C22–O23	125.9452	126.0233	126.1303	125.8856
C17–C22–O24	111.8693	111.6915	111.4631	111.662
O23–C22–O24	122.1422	122.2492	122.3528	122.3859
C22–O24–H25	108.9627	108.9006	107.3244	106.9818
**Dihedral angle (°)**				
C6–C1–C2–C3	0.1387	-0.0658	-0.1179	-0.1134
C6–C1–C2–H7	-179.5828	179.4582	179.3357	179.3365
O10–C1–C2–C3	179.8735	-179.7928	-179.7204	-179.7137
O10–C1–C2–H7	0.152	-0.2688	-0.2669	-0.2638
C2–C1–C6–C5	-0.1631	0.2533	0.2802	0.2654
C2–C1–C6–O12	179.572	-179.6696	-179.5901	-179.5996
O10–C1–C6–C5	-179.9136	179.9955	179.9066	179.8897
O10–C1–C6–O12	-0.1785	0.0726	0.0363	0.0246
C2–C1–O10–H11	-1.3775	2.1103	1.0867	0.9343
C6–C1–O10–H11	178.3608	-177.6196	-178.5211	-178.6709
C1–C2–C3–C4	0.1132	-0.248	-0.1454	-0.1077
C1–C2–C3–H8	-179.6566	179.1488	179.1466	179.1653
H7–C2–C3–C4	179.8338	-179.7698	-179.5944	-179.553
H7–C2–C3–H8	0.064	-0.373	-0.3023	-0.28
C2–C3–C4–C5	-0.3294	0.3601	0.2374	0.17
C2–C3–C4–C14	179.4364	-179.3201	-178.7735	-178.5415
H8–C3–C4–C5	179.4383	-179.0295	-179.0491	-179.0978
H8–C3–C4–C14	-0.7959	1.2904	1.9399	2.1907
C3–C4–C5–C6	0.3066	-0.1712	-0.0716	-0.0136
C3–C4–C5–H 9	-178.9624	179.4794	179.4516	179.5238
C14–C4–C5–C6	-179.4625	179.5105	178.9435	178.7019
C14–C4–C5–H 9	1.2686	-0.839	-1.5334	-1.7606
C3–C4–C14–H15	137.5224	-39.9185	-38.1749	-38.4112
C3–C4–C14–H16	20.016	-157.2835	-156.1408	-156.5267
C3–C4–C14–C17	-101.1485	81.1839	82.8167	82.6146
C5–C4–C14–H15	-42.7173	140.4106	142.842	142.9145
C5–C4–C14–H16	-160.2237	23.0456	24.8761	24.7991
C5–C4–C14–C17	78.6118	-98.487	-96.1664	-96.0596
C4–C5–C6–C1	-0.0613	-0.1359	-0.188	-0.2054
C4–C5–C6–O12	-179.7808	179.7828	179.6745	179.6516
H9–C5–C6–C1	179.2147	-179.7891	-179.7142	-179.7457
H9–C5–C6–O12	-0.5048	0.1296	0.1483	0.1113
C1–C6–O12–H13	-178.2399	178.1691	178.7497	178.6502
C5–C6–O12–H13	1.4854	-1.7511	-1.1155	-1.2095
C4–C14–C17–H18	-59.1741	-60.3975	-60.1423	-59.7376
C4–C14–C17–N19	65.0576	63.7649	63.6775	64.1218
C4–C14–C17–C22	-173.9291	-175.0801	-173.9132	-172.8663
H15–C14–C17–H18	62.9283	61.366	61.9237	62.4033
H15–C14–C17–N19	-172.84	-174.4715	-174.2565	-173.7373
H15–C14–C17–C22	-51.8268	-53.3166	-51.8472	-50.7254
H16–C14–C17–H18	178.8072	177.1473	177.59	178.1498
H16–C14–C17–N19	-56.9611	-58.6902	-58.5902	-57.9909
H16–C14–C17–C22	64.0521	62.4647	63.8191	65.0211
C14–C17–N19–H20	-57.9006	-60.7656	-62.8667	-63.1442
C14–C 17-N19-H21	-178.8105	179.395	177.8816	177.8814
H18–C17–N19–H20	64.2536	61.3465	58.2903	57.8263
H18–C17–N19–H21	-56.6562	-58.4929	-60.9613	-61.1482
C22–C17–N19–H20	-179.9672	177.0908	174.2604	173.7564
C22–C17–N19–H21	59.123	57.2514	55.0087	54.782
C14–C17–C22–O23	10.7744	9.507	15.5278	18.8558
C14–C17–C22–O24	-171.5895	-172.6458	-167.103	-164.0679
H18–C17–C22–O23	-106.3775	-107.497	-100.4609	-96.6285
H18–C17–C22–O24	71.2586	70.3502	76.9083	80.4477
N19–C17–C22–O23	133.1236	132.1398	138.5506	142.1262
N19–C17–C 22-O24	-49.2402	-50.013	-44.0802	-40.7975
C17–C22–O24–H25	-179.56	-179.6465	-178.9823	-178.5659
O23–C22–O24–H25	-1.8201	-1.7052	-1.4975	-1.3709

In the case of heteronuclear bond angle, such as C1–O10–H11, C6–O12–H13, H15–C14–H16, C17–N19–H20, C17–N19–H21, H20–N19–H21, O23–C22–O24 and C22–O24–H25 were found to be enlarged and was ranged from 2.182° to 3.169°. These bond angle changes were mainly due to electronic, Space Charge and oriental polarization taking place in different entities of the molecular sites. In the case of chain, the orientation of molecular dipoles were taking place, in the case of hexagonal ring, the separation or distortion of electron cloud around molecules with respect to the interactive and repulsive force constant. The movements of charges around molecular site were resulting in alignment of charge dipoles causing heterogeneous polarization which induced strong dielectric behavior of the material.

### 4.5 Mulliken charge assignment

**Figure 3:**
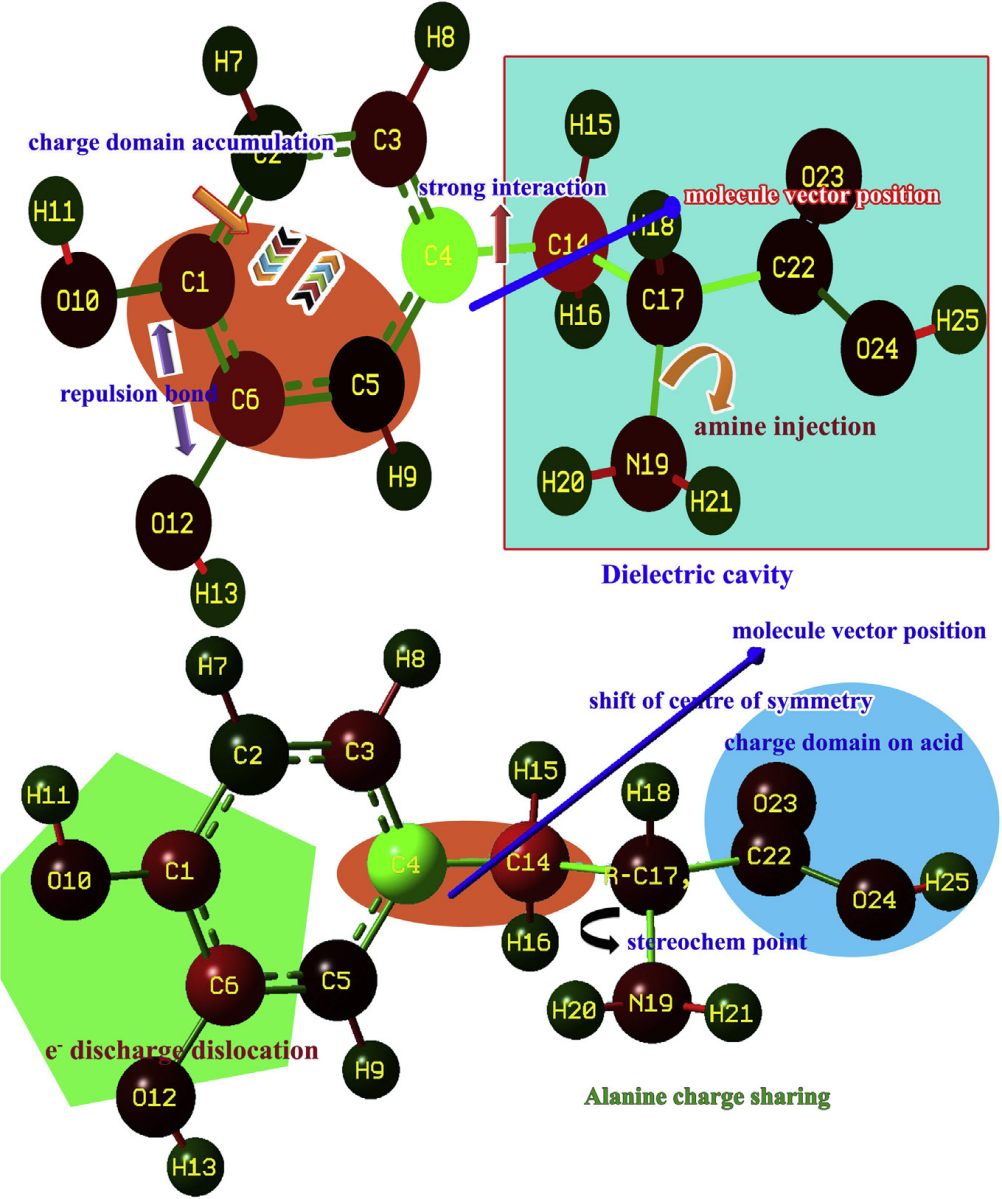
Mulliken charge distribution of 3-(3,4-Dihydroxyphenyl)-L-Alanine.

Linear combination of atomic orbitals (LCAO) system overlapped with one another with respect to the spatial quantization for formation of molecular orbitals and produced degenerate orbital interactions in which the dipole and electronic polarization were observed to explore the inducement of local electrical field by the applied electric field. The magnitude of local field can be modified by chemical potential generated around the molecular structure in the crystal which is quite significantly the space and electronic polarization of structure arrangement. The invariable mulliken charge assignment is exhibited in Figure 3.

Here, the charges on molecular site were found to be reoriented with respect to the local field and such that the negative charges from hydroxyl groups were delocalized and moved towards the core CC of benzene ring and shifted to the chain via C4. The electron cloud was populated on C1, C3 and C6 and they drained along the chain. Similarly, the charge domain was getting moved from the acid group and displaced towards C14 at which the charged domain was repelled and thus the charged domains depleted and formed boundary between them. The depletion force existed between domains leading Lorentz force which usually acted on dielectric cavity in the organic composite crystal. When the field is applied, the local electric field was induced and was distinguished from entities to entities in crystal. The well distinct domains were distributed over the material such that formed dielectric cavities which is able to process the high degree of birefringence gradient.

**Figure 4:**
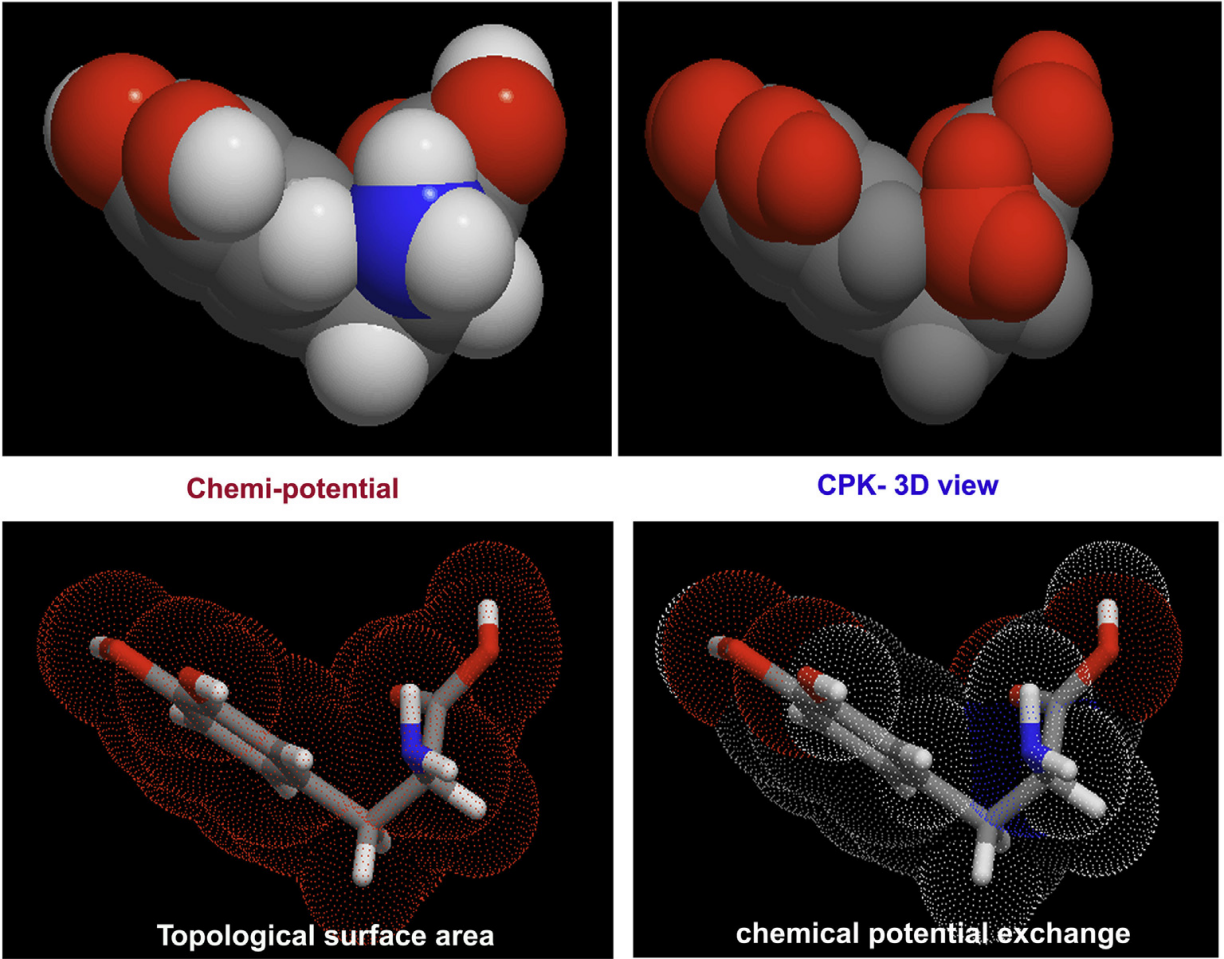
Topological surface view of 3-(3,4-Dihydroxyphenyl)-L-Alanine.

In this case, there were 16 heteronuclear and 8 homonuclear bonds appeared in which the dipoles formed electronic polarizability and homonuclear bonds created space charge polarization. At this juncture, the heteronuclear bonds C1–O10, C6–O12, C17–N19, C22–O23 and the heterogeneity was produced even on homonuclear bonds such as C4–C14, C2–C3 and C3–C4 by the charge delocalization due to space charge polarization and these were produced the strong σ and π-conjugative interactions which persuade spontaneous optical axes in different coordinates that making different refractive indices in the organic composite material. The composition of chemi-potential on the molecular structure was illustrated in Figure 4. According to the hyper-chemical calculation, the hyper and hypo-chemical potential was demonstrated by the red and blue colour gradient respectively. The molecular structure was appeared to be chair form and folded view showed helical manifestation and the topological surface area was drawn by electrostatic field grid points around the compound. The parametric potential oscillation was seen between ring and chain to explore resultant chemical hardness gradient.

### 4.6 Vibrational analysis

#### 4.6.1 Vibrational assignment

**Figure 5:**
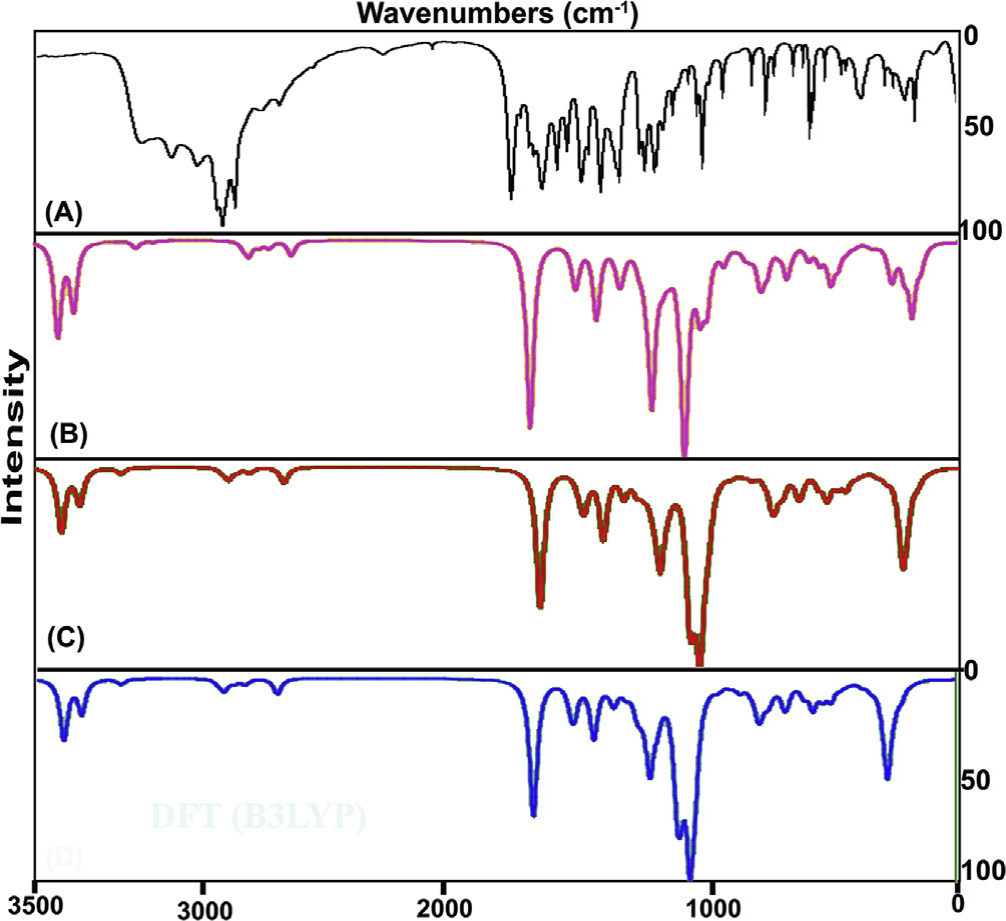
Experimental and calculated FT-IR spectra of 3-(3,4-Dihydroxyphenyl)-L-Alanine.

**Figure 6:**
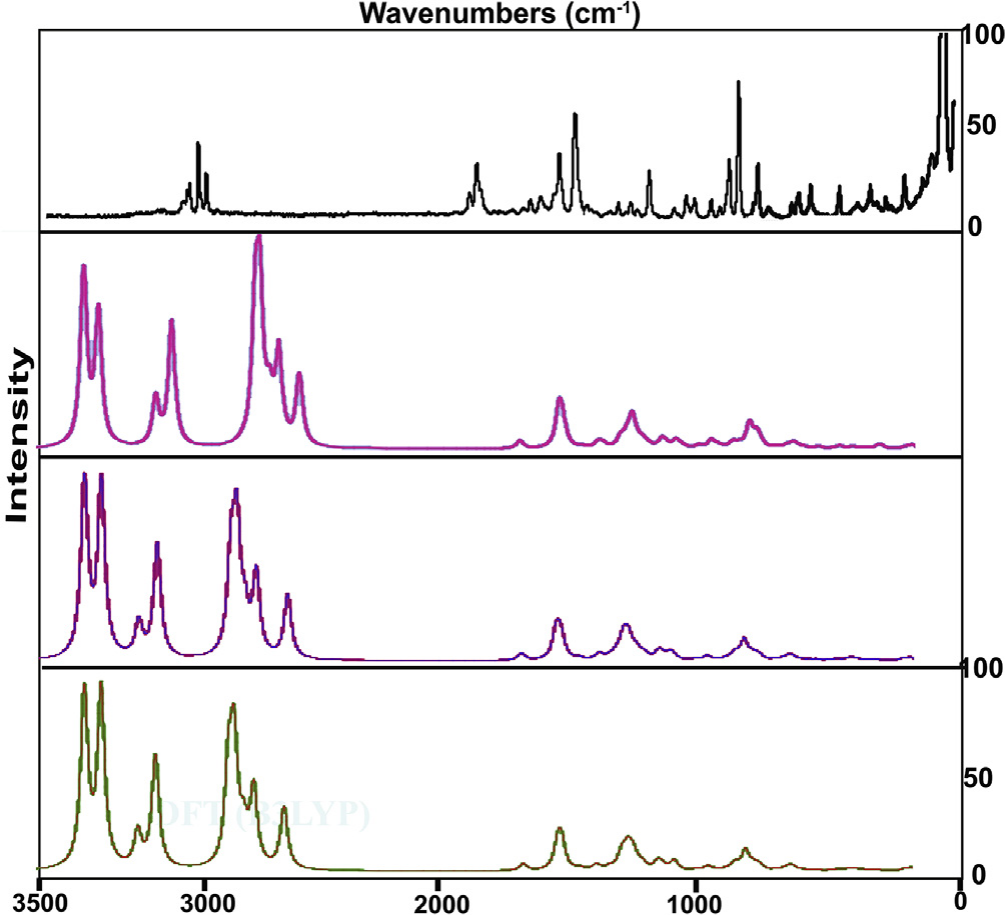
Experimental and calculated FT-Raman spectra of 3-(3,4-Dihydroxyphenyl)-L-Alanine.

The title composite was fabricated by amino acid species and organic compound by which the vibrational pattern was recorded and was synchronized with calculated wavenumbers. The finger print and group frequencies were assigned with respect to the characteristic vibrational regions and mutual exclusion principle. The obtained frequency pattern along with simulated spectra in terms of IR and Raman bands are illustrated in Figures 5 and 6 respectively and the related IR and Raman assignment are depicted in Table 4. As per the selection rule and point group of symmetry (C_S_), the in plane and out of plane vibrations were calculated to be 69 in which the in plane and out of plane vibrations are classified as 48 and 23 respectively. In the observed wavenumbers, the peaks were obtained with from very strong to weak intensity according to the force constant of bonds.

**Table 4:** Experimental and calculated vibrational frequencies of 3-(3,4-Dihydroxyphenyl)-L-Alanine.

S. No	Symmetry Species C_s_	Observed Frequency(cm-^1^)		Methods				Vibrational assignments
				HF	B3LYP		B3PW91	
		FT-IR	FT-Raman	6-311++G (d, p)	6-31+G (d, p)	6-311++G (d, p)	6-311++G (d, p)	
1	A**′**	3400m	-	3615	4043	3655	3666	(O–H) υ
2	A**′**	-	3395vw	3614	4039	3651	3663	(O–H) υ
3	A**′**	3290m	-	3268	3971	3580	3591	(O–H) υ
4	A**′**	3280m	-	3056	3693	3419	3426	(N–H) υ
5	A**′**	3240m	3240w	2985	3617	3342	3344	(N–H) υ
6	A**′**	3030m	-	2896	3212	3022	3018	(C–H) υ
7	A**′**	3010w	3010w	2869	3191	2997	2994	(C–H) υ
8	A**′**	-	3005m	1673	3188	2995	2990	(C–H) υ
9	A**′**	2990m	2990w	1653	3142	2954	2957	(C–H) υ
10	A**′**	2985m	2985w	1629	3098	2911	2907	(C–H) υ
11	A**′**	-	2930vw	1573	2999	2778	2769	(C–H) υ
12	A**′**	1650vs	-	1592	1931	1729	1739	(C=O) υ
13	A**′**	1610m	1610vw	1564	1743	1579	1586	(C=C) υ
14	A**′**	1590m	-	1551	1731	1571	1570	(C=C) υ
15	A**′**	1575vw	-	1545	1724	1561	1558	(C=C) υ
16	A**′**	1570vw	-	1652	1635	1482	1485	(O–H) δ
17	A**′**	1520w	-	1561	1552	1400	1403	(O–H) δ
18	A**′**	1500m	-	1547	1536	1400	1395	(O–H) δ
19	A**′**	1495m	-	1535	1522	1351	1349	(N–H) δ
20	A**′**	1460m	-	1455	1443	1307	1322	(N–H) δ
21	A**′**	1440s	1440vw	1427	1415	1296	1290	(C–C) υ
22	A**′**	1410vw	1410vw	1422	1410	1283	1277	(C–C) υ
23	A**′**	1405vw	-	1399	1387	1270	1270	(C–C) υ
24	A**′**	1365w	-	1374	1366	1252	1254	(C–O) υ
25	A**′**	1360w	-	1353	1341	1238	1233	(C–O) υ
26	A**′**	-	1350vw	1345	1334	1223	1222	(C–O) υ
27	A**′**	-	1330w	1318	1311	1206	1197	(C–C) υ
28	A**′**	1300m	-	1729	1249	1142	1138	(C–C) υ
29	A**′**	1295m	1295w	1725	1246	1140	1121	(C–C) υ
30	A**′**	1280w	-	1247	1239	1125	1121	(C–H) δ
31	A**′**	-	1270vw	1233	1224	1121	1119	(C–H) δ
32	A**′**	1265w	-	1638	1179	1088	1097	(C–H) δ
33	A**′**	1260w	-	1621	1169	1068	1080	(C–H) δ
34	A**′**	1220vs	-	1595	1141	1065	1063	(C–H) δ
35	A**′**	1205vs	-	1474	1066	973	972	(C–H) δ
36	A**″**	1170vs	1170vw	1412	1012	921	922	(N–H) γ
37	A**″**	-	1130vw	1404	1004	885	887	(N–H) γ
38	A**″**	1120w	-	1344	972	880	874	(O–H) γ
39	A**″**	1070vs	1070vw	1315	948	840	837	(O–H) γ
40	A**″**	-	990vw	1261	908	811	808	(O–H) γ
41	A**″**	-	985vw	1239	897	798	795	(C–H) γ
42	A**″**	980vs	-	1208	872	769	766	(C–H) γ
43	A**″**	950vs	950vw	1141	821	755	757	(C–H) γ
44	A**″**	-	945vw	1092	787	722	722	(C–H) γ
45	A**″**	920vs	920vw	1079	785	700	699	(C–H) γ
46	A**″**	-	915vw	1049	768	686	685	(C–H) γ
47	A**′**	880vs	880vw	948	687	622	619	(CCC) δ
48	A**′**	-	875vw	884	639	585	584	(CCC) δ
49	A**′**	840vs	840vw	845	609	560	558	(CCC) δ
50	A**″**	820s	820vw	817	588	544	541	(CCC) γ
51	A**″**	-	815vw	774	557	510	508	(CCC) γ
52	A**″**	780vs	780m	713	520	471	469	(CCC) γ
53	A**′**	-	775m	681	491	445	441	(C–O) δ
54	A**′**	750vs	-	667	482	434	432	(C–O) δ
55	A**′**	740vs	-	573	410	384	384	(C–O) δ
56	A**′**	720vs	720vw	535	382	347	348	(C–C) δ
57	A**′**	-	715vw	696	328	302	302	(C–C) δ
58	A**′**	-	680vw	686	322	297	295	(C–C) δ
59	A**″**	-	620vw	434	313	284	282	(C–O) γ
60	A**″**	-	590vw	410	289	277	274	(C–O) γ
61	A**″**	-	560vw	570	260	266	264	(C–O) γ
62	A**″**	-	470vw	347	225	249	257	(C–C) γ
63	A**″**	410w	-	332	198	221	220	(C–C) γ
64	A**″**	390w	-	278	186	181	179	(C–C) γ
65	A**′**	340w	-	235	167	150	149	(CNH_2_) δ
66	A**′**	300w	-	300	68	61	61	(CCOOH) δ
67	A**′**	220w	-	218	58	50	49	(CCH_2_) δ
68	A**″**	190w	-	192	49	26	43	(CNH_2_) γ
69	A**″**	120w	-	120	32	26	23	(CCOOH) γ

VS –Very strong; S – Strong; m- Medium; w – weak; as- Asymmetric; s – symmetric; υ – stretching; α –deformation, δ – In-plane-bending; γ-out-plane- bending; τ – Twisting:

#### 4.6.2 O–H vibrations

In the present case, the O–H is present in phenol as well as acid groups at which the O–H vibrations may be differed between two groups. In the absence of intra-molecular hydrogen bonding, phenols have an absorption band at 3400-3240 cm^−1^ for O–H stretching, at 1350-1200 cm^−1^ for O–H in plane bending and at 720-600 cm^−1^ [[16], [17]]. According to the Table 4, the O–H stretching, in plane and out of plane bending modes were observed at 340, 3395 & 3210 cm^−1^, 1570, 1520 & 1500 cm^−1^ and 1120, 1070 & 990 cm^−1^ respectively for the current case. Except stretching, all the observed wavenumbers were found to be elevated well above the expected region which was due to the chemical energy reservoir at phenol group. Here, the O–H bond in two different places was greatly pronounced at prominent place of vibrational band.

#### 4.6.3 COOH vibrations

The acid group is placed on the amino acid chain which very dominant usually and play important role in activation of vibrational activity of the molecule and in that way, the structural significance is exposed in the molecular structure. In amino acid, the C=O stretching band usually allotted in the region 1680-1650 cm^−1^ [18]. In this acid adopted molecule, the C=O stretching mode was recognized with very strong intensity at 1650 in IR spectrum which represent acid group in the spectra very consistently.

The C–O stretching vibration for mono and di-substituted phenols is observed in the region of 1300-1200 cm^−1^. The connected in plane and out of plane bending modes are usually found in the region 450-375 cm^−1^ and below 350 cm^−1^ respectively [19]. here, C–O stretching, in plane and out of plane deformation was identified with medium intensity at 1365, 1360 & 1350 cm^−1^, 775, 750 & 740 cm^−1^ and 620, 590 & 560 cm^−1^ respectively. The phenol vibrations were supported here by adjoining nodal group of phenol.

#### 4.6.4 Amino group vibrations

In primary amino acid molecule, typically N–H stretching vibrations suitably take place in the region 3500-3300 cm^−1^ [20]. The NH_2_ group has normally classified in to two vibrations; asymmetric and symmetric mode of vibrations. The asymmetric frequency is always elevated than symmetric and such asymmetric and symmetric N–H stretching vibrations of were acknowledged at 3280 cm^−1^ and 3240 cm^−1^respectively for this case. In addition to that, the asymmetric mode is always more intense than symmetric whereas here, both were observed with medium intensity. The N–H in plane bending (scissoring mode) are regularly found in the region 1610-1630 cm^−1^, rocking signal is assigned in the range 1100–1200 cm^−1^ and the wagging and twisting (out of plane bending) vibrations are recognized under 900 cm^−1^ [21]. In this molecule, the N–H in plane bending vibrations as scissoring mode was looked at 1495 and 1460 cm^−1^. Relatively, N–H out of plane bending as wagging mode have been assigned at 1170 and 1130 cm^−1^. As per the expected region of N–H modes, all the bending vibrational bands were recognized to be moved up to the higher region of spectrum due to the consistency of dipole bonds and it involved in the molecular typical character and participated in the hyper active polarization taking place in the molecule.

#### 4.6.5 C–H vibrations

The phenol derivatives usually having stretching bands of C–H bonds arranged in the region 3100-3000 cm^−1^ and such vibrational region may be infected by the addition of substitutions in the hexagonal ring. Associated in plane and out of plane bending vibrational bands are assigned as usual manner in the region 1300-1000 cm^−1^ and 950-809 cm^−1^ respectively [22, 23]. The present case was having stretching and in plane and out of plane deformation bands have been assigned at 3030, 3010 & 3005 cm^−1^, 1280, 1270 & 1265 cm^−1^ and 985, 980 & 950 cm^−1^ in that order. All the C–H vibrations have been found well within the expected region. Except substitutions at core, three C–H bonds have been identified by the observation of vibrational characteristics. This vibrational process of allied dipole bonds are concerned in the molecular property in physical sense.

In the case of aliphatic chain, the C–H stretching vibrations of the same is usually arranged in the region 3000-2800 cm^−1^ [24]. The related in plane and out of plane bending vibrations are normally observed in the range 1260-960 cm^−1^ and 900-670 cm^−1^ correspondingly [25]. At this time, the stretching, bending vibrations were particularly assigned at 2990, 2985 & 2930 cm^−1^, 1260, 1220 & 1205 cm^−1^ and 945, 920 & 915 cm^−1^ respectively for the present crystal compound. The stretching and in plane bending signals were obtained within the expected limit whereas the out of plane bending modes were observed well above the allowed region which indicates that, the vibrational energy have not affected by allied bonds and the consistent participation in crystal property.

### 4.7 NMR examination

**Figure 7:**
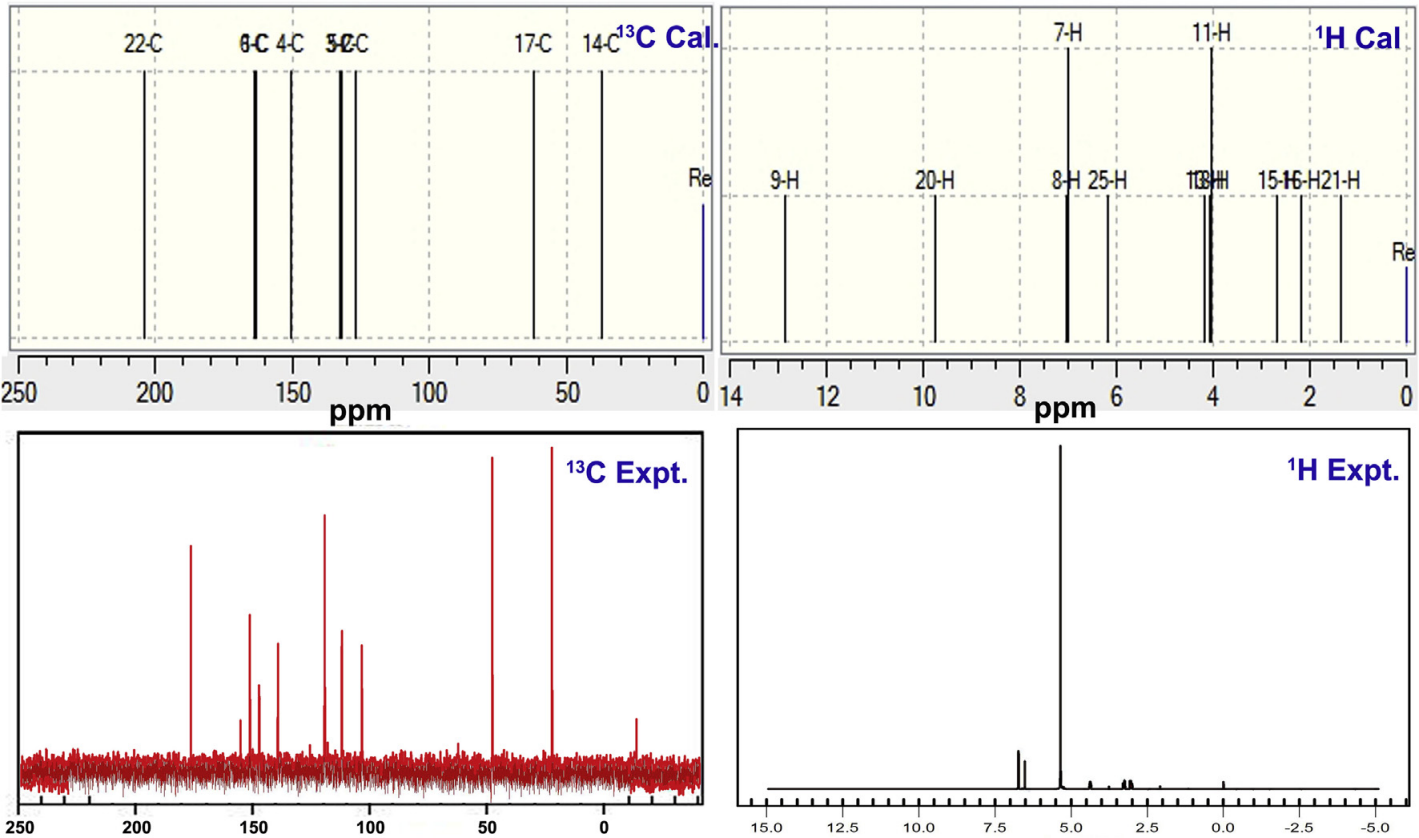
Experimental and calculated NMR spectra of 3-(3,4-Dihydroxyphenyl)-L-Alanine.

In the molecular orbital formation, electron cloud related to the atom is usually redistributed with respect to the chemical equilibrium forces among the molecular sites. The polarization of charges in both form among the molecular site are concentrated normally in electronegative and protopositive regions. In this fashion, the electron clouds are forced to be delocalized and associated chemical shift is totally modified. For the current case, the chemical shift is monitored in Table 5 and the corresponding experimental and simulated spectra are demonstrated in Figure 7. Due to the solvent and state of the crystal sample, the recorded and calculated chemical shifts were differed and they were corrected as per the correction factors.

**Table 5:** Experimental and calculated ^1^H and ^13^C NMR chemical shift in 3-(3,4-Dihydroxyphenyl)-L-Alanine.

Atom position	Chemical Shift - TMS-B3LYP/6-311+G(2d,p) (ppm)			Experimental shift (ppm)
	Gas	Solvent phase		
		DMSO	Water	
C1	163.348	163.374	163.348	155
C2	126.863	126.837	126.863	121
C3	132.365	132.347	132.365	128
C4	150.655	150.653	150.655	151
C5	132.02	131.997	132.02	138
C6	164.074	164.099	164.074	158
C14	37.2588	37.258	37.2588	32
C17	61.9095	61.9053	61.9095	58
C22	204.081	204.033	204.081	-
H7	7.00041	6.9944	7.0004	6.8
H8	7.00041	6.9944	7.0004	6.8
H9	12.8702	12.8702	12.8702	-
H11	4.04909	4.0372	4.0491	4.3
H13	4.20945	4.1946	4.2094	4.3
H15	2.68925	2.6875	2.6893	2.9
H16	2.18612	2.1899	2.1861	-
H18	4.04909	4.0372	4.0491	4.3
H20	9.74455	9.7438	9.74455	-
H21	1.3525	1.3443	1.352	-
H25	6.20583	6.1963	6.2058	6.0

Generally, in aromatic case with substitutions, the chemical shift of core carbons of ring is always observed greater than 100 ppm [26]. In this case, the benzene was triply substituted hexagonal ring in which, except C1, C4 and C6, all the core carbons like C2, C3 and C5 were shifted chemically by 126, 132 and 131 ppm (Expt.- 121, 128 and 138 ppm) respectively. The chemical shift was comparatively high since core carbons positioned around the substitutional place. The chemical shift of C1, C4 and C6 were observed to be 163, 150 and 164 ppm (Expt.- 155, 151 and 158 ppm) respectively. The chemical shift of C1 and C6 was found to be high which was mainly due to the hydroxyl group where the paramagnetic shield was broken randomly since they acted as chemical nodal region of hexagonal ring. At C4, the electron delocalization taking place due to the bridge point of electron cloud harmonic oscillation. The C14 and C17 were determined to be having small chemical shift of 37 and 61 ppm and this was purely due to the intermediate allied carbons where during the chemical energy oscillation, electron cloud was virtually filled and simultaneously, paramagnetic shield was concealed and protected. The C22 was calculated to have large chemical shift of 204 ppm where the acid group pulled the electron cloud and send them in to the ring via chain. The charge oscillation restricted point was found to be acid group, from which the electron cloud was partially discharged towards the bridge point C of ring. In this case, the chemical shift clearly elucidated that, the oscillating chemical potential movement was observed among core carbons of hexagonal ring and bridge carbons of chain. The resultant oscillating charge cloud was strongly showed the chemical potential which leads the chemical kinetics in the molecular structure and thereby crystal.

### 4.8 Frontier molecular orbital interaction study

**Figure 8:**
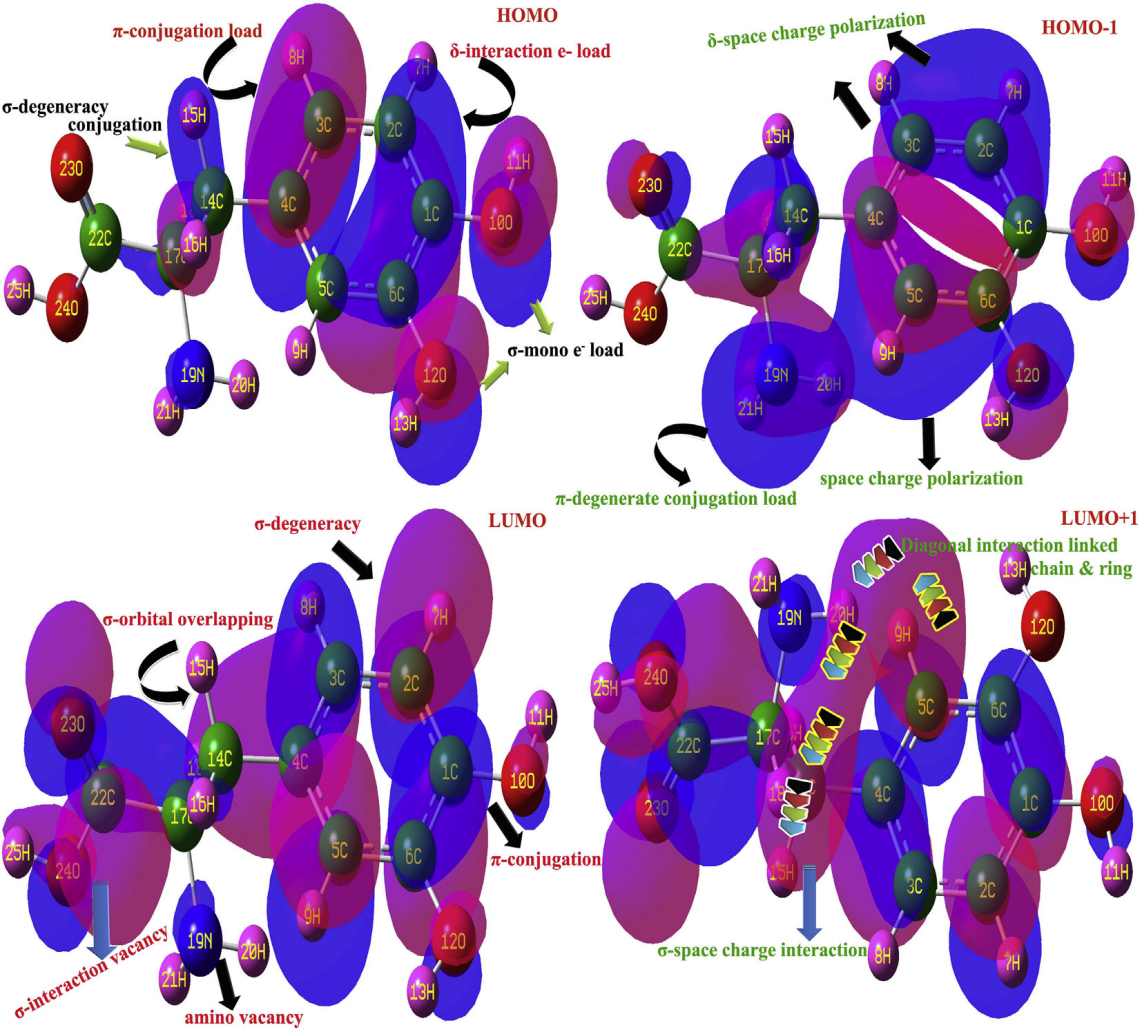
Frontier molecular orbital interaction of 3-(3,4-Dihydroxyphenyl)-L-Alanine.

Usually, the chemical energy states in the form of electronic energy levels are splitted up in to two major orbital levels such as HOMO and LUMO among which the transitions between the corresponding energy levels with respect to the frontier selection rule. The chemical kinetics in the organic crystal for producing desired properties is usually restored in such transitions. The chemical potential also stored in the compound by quantized disintegrates molecular spatial orbitals in terms of electronic energy. The reserved chemical energy can be recognized by identical σ, π and δ-conjugation orbital interaction and they are shown in Figure 8 and their respective energy profile values are portrayed in Table 6.

**Table 6:** Frontier molecular orbitals with energy levels 3-(3,4-Dihydroxyphenyl)-L-Alanine.

Energy levels	IR region		UV-Visible region
	B3LYP/6-311++G(d,p) Energy (eV)	B3PW91/6-311++G(d,p) Energy (eV)	
H+10	10.7613	10.7309	
H+9	10.3406	10.2766	
H+8	10.1686	10.0881	
H+7	9.8170	9.6252	
H+6	9.6731	9.3544	
H+5	9.3359	9.2595	
H+4	9.1011	8.9112	
H+3	8.1667	8.1242	
H+2	7.0687	7.2611	
H+1	6.8965	6.9409	
H	6.0458	6.5701	
L	0.6833	1.3091	
L-1	0.5826	1.0432	
L-2	0.4131	0.6305	
L-3	0.3720	0.6259	
L-4	0.2490	0.3780	
L-5	0.0816	0.3532	
L-6	0.1518	0.1059	
L-7	0.3687	0.3687	
L-8	0.5883	0.4993	
L-9	0.7820	0.7333	
L-10	1.0174	1.0593	

In this case, the alanine and organic species were fused together formed semi-organic crystal where the orbital interaction take place impulsively with respect to the same energy orbital availability (degenerate orbitals). Here, in LUMO, the σ-orbital interaction processed fully over core carbons and partially over acid and hydroxyl groups where only σ-overlapping seems to be observed and they were fully empty with quantized amount of energy and able to receive the transitional energy in terms of electron. As per the LUMO+1, the space charge interactive empty orbitals were available to accept electronic energy and here, the σ and π-orbitals were interacted by space charge polarization and chain and ring reserved its own. These are direct acceptor interactive orbitals and quantized to allowed chemical potential as splitted electronic transitions.

The HOMO was appeared as π and δ –interactive system of orbitals concentrated on semicircle core carbons of hexagonal frame. Here, all the interactive orbitals are appeared to be merged by overlapping and all electrons in such orbitals are clubbed together and able to make resultant transitions to all available unoccupied orbitals. In this HOMO, all were seems to be π and δ-conjugated complex orbitals where the energy of all possible excited molecular energy states were blended and thereby the new properties are exposed from the product organic crystal. In HOMO-1, the space charge polarization made all excited orbitals of all core carbons and part of chain together which was observed to be more intense than HOMO. So it was inferred that, when going from lower order orbital to higher level, the degenerate orbital interaction increased more and more and finally all orbitals of particular energy are linked and thus, the peculiar properties were persuaded. Accordingly, all types of higher order and lower order orbital interactions in this compound made electro-optical properties. The energy gap of present case in first order and second order were 5.26 and 5.36 eV correspondingly which confirmed high degree of dielectric constant of present organic crystal and simultaneously the present case possessed SHG and THG characteristics.

### 4.9 UV-visible absorption/transmission analysis

**Figure 9:**
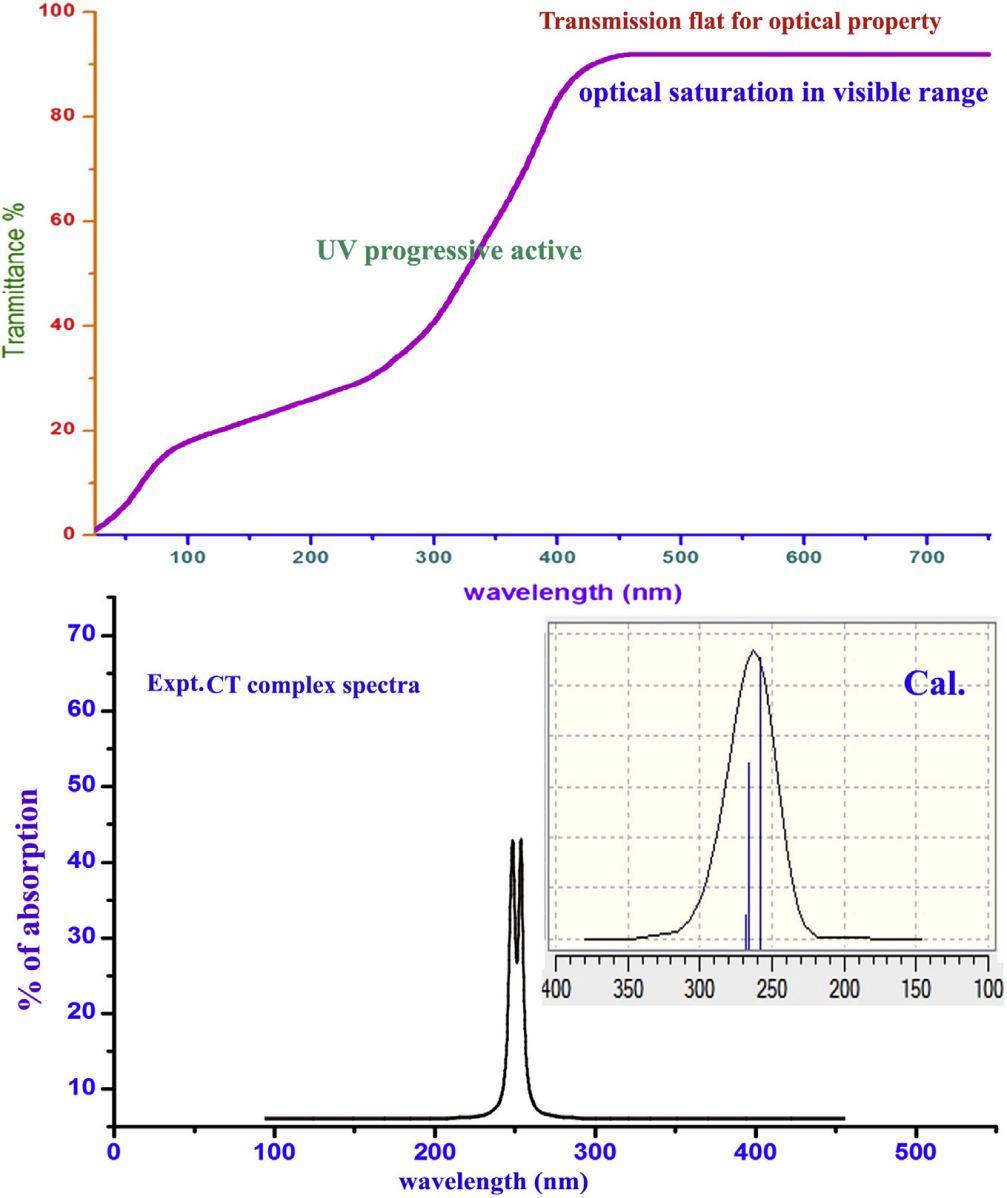
UV-Visible and transmission spectra of 3-(3,4-Dihydroxyphenyl)-L-Alanine.

The UV-Visible absorption spectral simply described existing electronic energy levels and their important transitions associated with Charge transfer complex causing fundamental properties of the organic complex [27] whereas UV-visible transmission spectral pattern used for investigating linear optical characteristics of the chemical compound. Here, both absorption and transmission curves are obtained for the present organic complex and are shown in Figure 9. The electronic absorption parametric values are portrayed in Table 7.

**Table 7:** Theoretical electronic absorption spectral values of 3-(3,4-Dihydroxyphenyl)-L-Alanine.

λ (nm)	E (eV)	(*f*)	Transition levels	Major contribution	Assignment	Region	Bands
**Gas**							
268.46	4.6183	0.0057	H+1→L(42%) H→L(-38%)	H+1→L(42%)	n→π*	Quartz UV	R-band (German, radikalartig)
265.68	4.6667	0.0102	H→L(48%) H→L-1(21%)	H→L(48%)	n→π*		
257.58	4.8134	0.0133	H→L(-32%) H→L-1(57%)	H→L-1(57%)	n→π*		
**DMSO**							
269.02	4.6087	0.0028	H+2→L(36%) H+1→L(49%)	H+1→L(49%)	n→π*	Quartz UV	R-band (German, radikalartig)
264.49	4.6878	0.0077	H+1→L(21%) H→L(60%)	H→L(60%)	n→π*		
257.37	4.8173	0.0256	H+1→L-2(23%) H→L-1(59%)	H→L-1(59%)	n→π*		
**Water**							
268.96	4.6098	0.0026	H+2→L(36%) H+1→L(49%)	H+1→L(49%)	n→π*	Quartz UV	R-band (German, radikalartig)
264.40	4.6892	0.0070	H+1→L(21%) H→L(61%)	H→L(61%)	n→π*		
257.26	4.8194	0.0241	H+1→L-2(24%) H→L-1(59%)	H→L-1(59%)	n→π*		

According to the table, three excited transitions observed at 268, 265 and 257 with the energy gap of 4.61, 4.66 and 4.81 eV at oscillator strength of 0.005, 0.01 and 0.013 respectively. All those transitions were assigned to n→π* system in Quartz UV region with the band of R-Band (German, radikalatig). These transitions are observed in real molecular phase and all those values are repeated in solvent phase also that confirmed solvent has taken no effect on absorption spectra. According to the assigned wavelengths of transitions, the CT of the present case was identified to be COOH group. Even though, partial transitions represent as H→L-1, H+1→L, H+2→L and H+1→L-2 which are belong to n→σ* and σ- σ* interactive orbital systems. The resultant electronic absorption was represented by transitions among three vibrational energy states. The UV-Visible energy gap was found to be 4.6–4.8 eV invariably and it is controlled by applied electric potential.

The UV-Visible transmission curves were observed to be flat from 400 nm to 700 nm which showed the good optical response of the present organic composite. The optical transmission response is also recognized even below 400 nm which described that, the present case able to reproduce UV signals with more than 60% intensity. If, the distinct wavelengths are looked out, the absolute optical response for actual wavelength will be determined. From the above observation, it is clear that, the present case is able to produce even laser if it is properly electronically pumped.

### 4.10 Physico-chemical parameters

As in Table 8, the zero point vibrational energy state of this case was found to be 705 Hartree in both regions since there was no structural difference observed between two regions. The resultant dipole moment of molecule; 3-(3,4-Dihydroxyphenyl)-L-Alanine was 2.22 and 3.20 dyne in IR and UV region respectively. Here, dipole moment was higher in UV than IR since the optical polarizability was greater in present case than electronic polarizability. The electron affinity and ionization potential was found to be 6.0459 eV & 0.6833 eV and 5.261 eV & 1.309 eV in IR and UV-Visible region in that order. Here, the forbidden energy gap formed in IR was greater than UV-Visible region that ensured current crystal was UV-Visible active.

**Table 8:** Physico-chemical parameters of 3-(3,4-Dihydroxyphenyl)-L-Alanine in UV-Visible region.

Parameter	B3LYP/6-311++G (d,p)	UV Visible	Electrophilicity charge transfer (ECT) = (ΔNmax)_A_-(ΔNmax)_B_
E_total_ (Hartree)	-705.4485	-705.1328	
E_HOMO_ (eV)	6.0459	6.5701	
E_LUMO_ (eV)	0.6833	1.3091	
ΔE_HOMO-LUMO gap_ (eV)	5.3626	5.261	
E_HOMO-1_ (eV)	6.8966	6.9409	
E_LUMO+1_ (eV)	0.5826	1.0432	
ΔE_HOMO-1-LUMO+1 gap_ (eV)	6.314	5.897	+0.0918
Chemical hardness (η)	2.6813	2.6305	
Electronegativity (χ)	3.3646	3.9396	
Chemical potential (μ)	3.3646	3.9396	
Chemical softness(S)	10.7252	10.522	
Electrophilicity index (ω)	2.111	2.9501	
Dipole moment	2.2261	3.2041	
ETC	2.6191	2.4649	

The Chemical hardness (η) of the organic composite reflect the chemical inertness of crystal and here it was 2.68 which was moderate and present organic composite was rather hard to receive other chemical species to fuse. The Chemical softness(S) of this case determined to be 10.7252 which were large and it was enough to accept further organic or inorganic species to produce new compound and thereby properties of the product is different. The Electronegativity (χ) is used to measure the electronegative characteristics of organic complex and here, the same was found to be 3.3646 and 3.9396 in IR and UV region respectively. In both regions, the specific parameter was traced to be high which showed heterogeneity atmosphere in charge levels and hence, it proved the existence strong polarizability. The Electrophilicity index (ω) of present composite was to be 2.11 and 2.95 in both regions and these values illustrated that, distributed anomalous charge domains were found to be present in different coordinates of the crystal composite. The ECT gradient was observed to be +0.0918 and it showed the chemical equilibrium of the compound by which it is acted as saturated chemical species.

### 4.11 NLO activity analysis

The non linear optical activity is usually customized by the inducement of frequency dependent polarization occurred in the crystal materials. The first order and second order polarizability was calculated and are presented in the Table 9. For this compound, the average and net polarizability were calculated in different coordinates of the molecular complex. It was found to be 83.79 in XX coordinate and it was represented as α_xx_. Similarly, the polarizability was found to be 74.56 in YY component and it was symbolized to be α_yy_. In ZZ component it was determined to be 82.71 denoted as α_zz_. Form these values, it is observed that, in three coordinates (X,Y,Z), the polarizability was taking place and hence such values offering different refractive indices for optical waves. Thus, the birefringence effect is ensured in the crystal composite. In addition to that, the total polarizability α_tot_ and average polarizability Δα are observed to be 168 × 10^−33^ esu and 225 × 10^−33^ esu respectively. These parameters were validated the hypo-polarizability in the material and thereby it was optically active. The hyper active polarizability is found to be 91.2 × 10^−33^ esu, 26.63 × 10^−33^ esu and 44.67 × 10^−33^ esu at xxx, xxy and xyy coordinates and these are represented as β_xxx_, β_xxy_ and β_xyy_ respectively. from this view, it was clear that, the hyper action first coordinate is more than rest of two coordinates. The resultant hyperpolarizability is calculated to be 1775.05 × 10^−33^ esu which is five times greater than hyperpolarizability of thio urea. So, in this case, the SHG and THG will be possible with maximum slew rate.

**Table 9:** The polarizability α(a.u.) and the first hyperpolarizability β(esu) of 3-(3,4-Dihydroxyphenyl)-L-Alanine.

Parameter	B3PW91/6-31++G(d,p)	Parameter	B3PW91/6-31++G(d,p)
α_xx_	83.790	β_xxx_	91.2781
α_xy_	8.890	β_xxy_	26.633
α_yy_	74.560	β_xyy_	29.739
α_xz_	-5.0881	β_yyy_	9.387
α_yz_	6.288	β_xxz_	44.675
α_zz_	82.7185	β_xyz_	-10.897
α_tot_	168.98	β_yyz_	-1.625
Δα	225.94	β_xzz_	-6.062
μ_x_	0.5994	β_yzz_	11.002
μ_y_	-0.6866	β_zzz_	-8.460
μ_z_	-1.9671	β	1775.058
μ	2.1679		

### 4.12 MESP analysis

**Figure 10:**
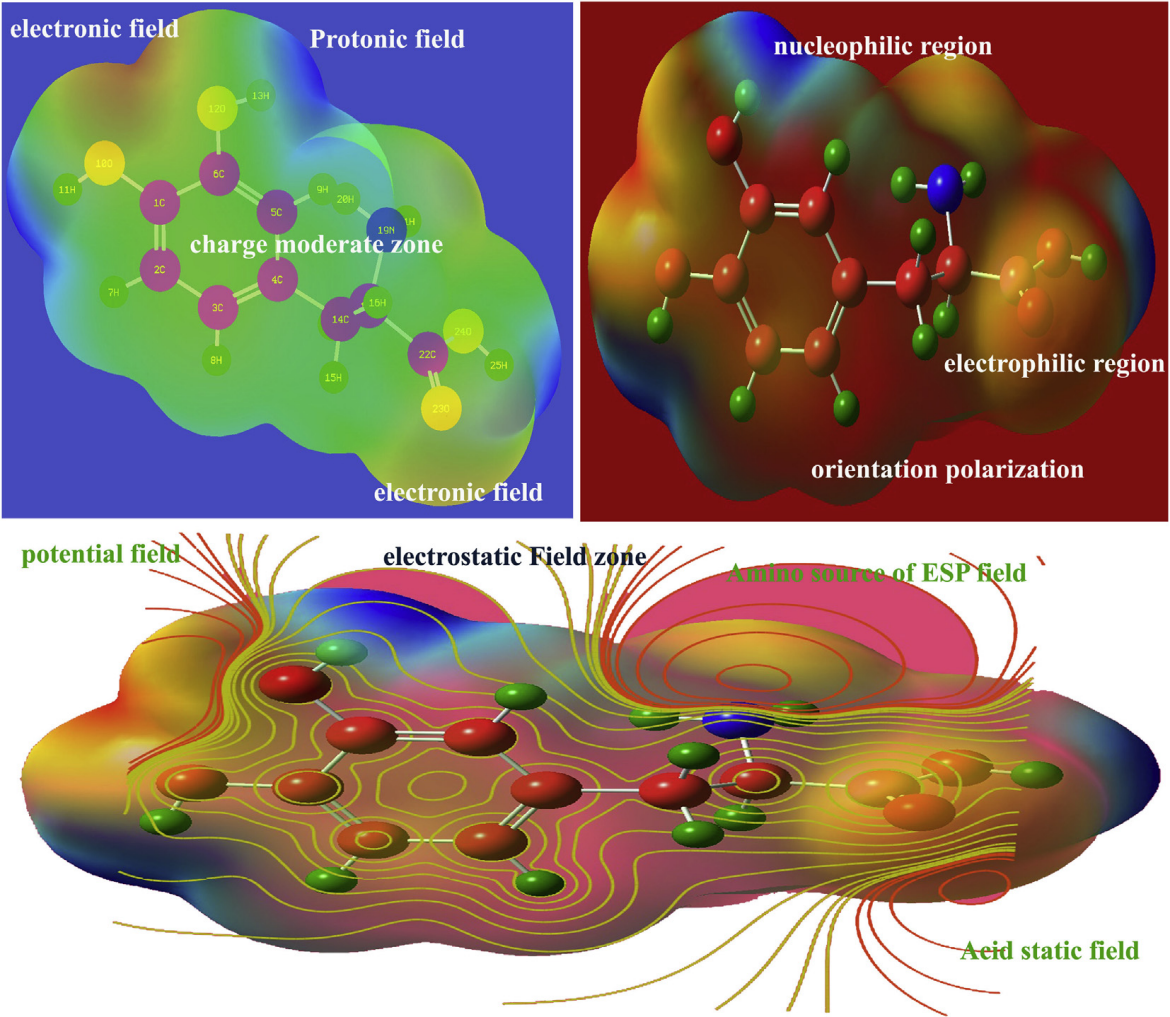
MEP display of 3-(3,4-Dihydroxyphenyl)-L-Alanine.

The electrostatic potential energy Laplacian distribution on the molecular complex showed the charge displacement due to the existence of depletion potential. The molecular charge delocalized according to the electronegative (electrophilic) and protopositive (nucleophilic) enforcement in the molecular site. It gives clear cut evidence of hypo and hyper polarization due to the homo and heteronuclear interactions and repulsions [28]. Here, the molecular structures are arranged periodically by performing translation symmetry operation in the crystal structure and hence, the two dissimilar charge domains in the molecular site were displaced and retained at particular places around the atoms that were displayed in Figure 10. After the completion of fusion of alanine and dihydroxybenzene, the molecular charges were settled down; this was rated by color gradient from red (electrophilic) to blue (nucleophilic).

Highly electrophilic zones were appeared over the OH and COOH groups in which the potential is measured to be -5.241 e^−2^. Extremely positive region known as protonic zones are found around H of hydroxyl, benzene ring and COOH groups in which the potential is calculated to be +5.241 e^−2^. The entire Laplacian charge distribution is controlled by ±5.241 e^−2^ by which the hyper and hypo-polarizability is determined. Here, the phenolic electrostatic field is graded lower than acidic static field and in this case, the phenol, amino group and acid groups are acted as main field source and from which the field is distributed. This entire electrostatic field of molecular structure formed the enantiometric scale that causing binding of molecular structures in the crystal.

### 4.13 NBMO studies

Except bonding orbitals in the molecule, all the orbitals called non bonding molecular orbitals and the quantized transitions among important entities are used to validate the chemical potential exchange to produce chemical, optical, electrical and biological properties [29]. The chemical potential energy was measured usually from the transitional energy which is directly proportional to the energy difference between two any orbitals with occupation energy [30]. Many transitions were observed in the interactive orbitals of present molecular complex of which some important transitions were presented in Table 10.

**Table 10:** The calculated NBO of 3-(3,4-Dihydroxyphenyl)-L-Alanine by second order Perturbation theory.

Donor (i)	Type of bond	Occupancy	Acceptor (j)	Type of bond	E2 kcal/mol	Ej – Ei au	F(I j) au
C1–C2	π	1.98366	C1–C6	π*	2.01	1.13	0.043
C1–C2	π		C6–O12	π*	2.34	1.02	0.044
C1–C2	π		C3–C4	π*	10.01	0.32	0.051
C1–C2	π		C5–C6	π*	11.07	0.30	0.053
C1–C6	σ	1.97055	C2–H7	σ*	2.54	1.09	0.047
C1–C6	σ		C5–C6	σ*	2.08	1.27	0.046
C1–C6	σ		C5–H9	σ*	2.08	1.27	0.046
C1–C10	σ	1.99292	C2–C3	σ*	2.13	1.24	0.046
C2–C3	σ	1.96996	C1–O10	σ*	5.32	0.87	0.061
C2–C3	σ		C3–C4	σ*	2.21	1.25	0.047
C2–C3	σ		C4–C14	σ*	4.68	1.00	0.061
C2–H7	σ	1.97894	C1–C6	σ*	5.23	0.91	0.062
C3–C4	π	1.98098	C4–C5	π*	2.19	1.14	0.045
C3–C4	π		C1–C2	π*	10.90	0.30	0.051
C3–C4	π		C5–C6	π*	9.93	0.29	0.049
C3–C4	π		C14–H16	π*	2.99	0.71	0.043
C3–H8	σ	1.97923	C4	σ*	2.16	1.63	0.053
C3–H8	σ		C4–C5	σ*	6.63	0.93	0.070
C5–C6	π		C4–C5	π*	2.62	1.16	0.049
C5–C6	π		C1–C2	π*	9.52	0.31	0.050
C5–C6	π		C3–C4	π*	11.75	0.33	0.056
C5–C9	σ	1.90624	C1–C6	σ*	4.92	0.92	0.061
C5–C9	σ		N19–H20	σ*	51.34	1.16	0.219
C17–H18	σ		C22–O23	σ*	4.47	0.52	0.045
N19–H20	σ	1.95392	C5–H9	σ*	20.07	1.21	0.139
O10	LP		C1–C2	π*	4.43	1.21	0.066
O10	LP		C1–C2	π*	17.78	0.36	0.074
O12	LP		C5–C6	π*	18.53	0.36	0.076
N19	LP	1.99937	C17–H18	σ*	6.45	0.74	0.062
O23	LP	1.99977	C22	N*	14.81	1.62	0.138
O23	LP		C22	N*	3.28	1.71	0.069
O23	LP		C17–C22	π*	13.18	0.62	0.082
O23	LP		C22–O24	σ*	30.57	0.52	0.113
O24	LP	1.99976	C22–O23	π*	5.55	1.12	0.071
O24	LP		C22–O23	π*	24.04	0.35	0.082
C1–C2	π	1.98366	C3–C4	π*	70.82	0.02	0.062
C5–C6	π	1.98162	C3–C4	π*	53.50	0.02	0.064

In the ring, the transitions were obtained from donor of (C1–C2) (occup. Energy-1.98366) to C3–C4 and C5–C6 by taking energy of 10.01 and 11.07 kcal./mol. at π- π* interactive systems. Similarly, from C3–C4(occup.energy-1.98098) to C1–C2 and C5–C6 by absorbing energy of 10.90 and 9.93 kcal./mol. at π- π* interactive system. The transitions from C5–C6 to C1–C2 and C3–C4 by consuming energy of 9.52 and 11.75 kcal./mol. in same π- π* interactive system. Other significant transitions were observed from C5–C9 to N19–H20 and reversely from N19–H20 to C5–H9 by taking energy of 51.34 and 20.07 kcal./mol. in σ – σ*. From the lone pair of O10 to C1–C2 and C5–C6 with the energy gap of 17.78 and 18.53 kcal./mol. another lone pair of O12 to C5–C6, O23 to C22, O23 to C17–C22 and C22–O24 by consuming energy of 18.53, 14.81, 13.18 & 30.57 kcal./mol. in n- σ*, n-n* and n- π* interacting systems of chain and ring. Consequently, from O24 to C22–O23, C1–C2 to C3–C4 and C5–C6 to C3–C4 by taking energy of 24.04, 70.82 and 53.50 kcal./mol in π- π* linking system. Most of the energy transactions were obtained from ring to chain and only less amount of energy observed from chain to ring. Large amount of energy was utilized for those transitions and generates chemical potential called activation potential that is the background of electronic controlled optical property inducement in the crystal material.

### 4.14 VCD and biaxial crystal analysis

Chirality is a physical property of the inorganic or organic molecule that results from its structure formation. The optical activity is directly recognized by identifying molecular structure in 3-D form in crystal materials. Normally, if the molecule is chiral, that molecule will be optically active and thus the formation of crystal is also optically dynamic [31]. The chiral like molecular compound is non-superposable on its mirror image which is usually formulated by the presence of asymmetric carbon nodal centre with electronegative groups of different masses. The vibrational circular dichroism techniques are not only used in biology for studying toxicity characteristics and also used to evaluate the optical properties in NLO crystallography and organo-metallic material research [32].

**Figure 11:**
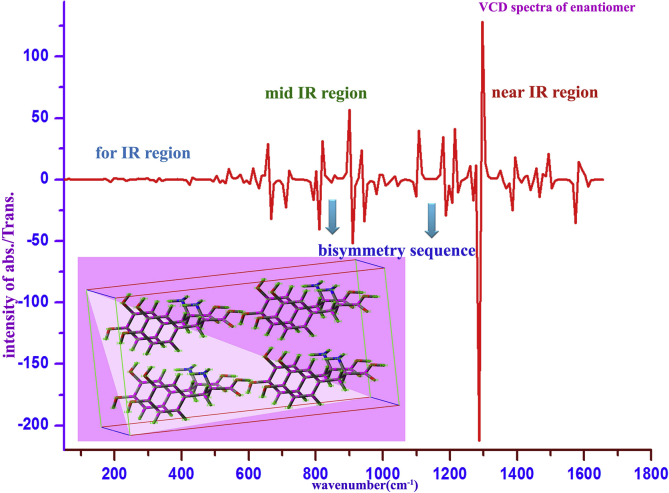
VCD spectra of 3-(3,4-Dihydroxyphenyl)-L-Alanine.

In this case, the L-Alanine has already having asymmetrical carbon centers for providing optical activity process in the molecule, and it was fused with dihydroxy benzene, so the optical nodal carbon centers were absolutely improved that guides enriched optical propagation with different velocities. It was validated by the observation of 3D molecular as well as crystal structures. As per Figure 11, the vibrational circular dichroic properties of the present compound were validated. The sequential arrangement of molecular structures within crystal material was detail shown in Figure where in which the vibrational circular characteristics have been monitored as per the far-IR, mid-IR and near-IR region along with Raman scattering transmission peaks.

According to the mutual exclusion principle, the active bands in IR were found to be weak in Raman which was validated by the observed VCD spectrum for this case. The alternative peaks in IR and Raman at far-IR were observed to be weak whereas in mid and near IR, the peaks were obtained with moderate intensity. This arrangement in the VCD spectrum ensured non-superimposable mirror-image forms of present molecular structures and here, optimized molecular geometry and associated symmetry point groups (C_S_) employed chirality functions that customized by uniquely associated with specific chiral chromophores present in the molecules. From this observation, it was concluded that, the present case was ensured to be biaxial symmetry crystal.

## 5 Conclusion

The organic-composite crystal; 3-(3,4-Dihydroxyphenyl)-L-Alanine, for optical applications was grown by slow evaporation method. The molecular (C_S_) and crystal symmetry (Lattice-orthorhombic) (Space group-*P*2_1_2_1_2_1_) were validated by examining the optimized molecular symmetry operation and XRD spectral pattern. The crystal parameters were evaluated and justified for the crystal physical chemical and structural properties. The formation of birefringence effect in the geometry of the crystal was confirmed by observing different refractive indices. The distortion of bond length and bond angles supported to dipole and oriental and electronic polarization for inducing non linear optical activity was proved. The asymmetric charge levels inside the molecule and crystal structure was monitored by the observation of mulliken charge assignment. The molecular heterogeneity was proved by the homo and heteronuclear bond orientation in the crystal compound. The anisotropic characteristics of present organic composite crystal were studied by the activeness of dipole and polarized bonds in different entities. The role compositional bonds in the crystal to customize the dielectric property were studied from the vibrational analysis. The chemical reaction path for finding the oscillation of chemical potential was monitored by the examination of chemical shift. The σ, π, δ- orbital conjugation interaction system in HOMO and LUMO arrangement was displayed and the exchange of interaction energy was studied and the linked energy gap mechanism was deliberated. The chemical parametric oscillation in terms of kinetic potential was measured from the non bonding molecular orbital interaction complex and maximum contribution of chemical energy to produce all properties was illustrated.

## Declarations

### Author contribution statement

D. Vidhya: Analyzed and interpreted the data.

S. Ramalingam: Conceived and designed the experiments; Wrote the paper.

S. Periandy: Contributed reagents, materials, analysis tools or data.

R. Aarthi: Performed the experiments.

### Funding statement

This research did not receive any specific grant from funding agencies in the public, commercial, or not-for-profit sectors.

### Competing interest statement

The authors declare no conflict of interest.

### Additional information

No additional information is available for this paper.
